# Development of Evidence-Based COVID-19 Management Guidelines for Local Context: The Methodological Challenges

**DOI:** 10.1155/2022/4240378

**Published:** 2022-04-20

**Authors:** Sarah Nadeem, Salima Saleem Aamdani, Bushra Ayub, Nashia Ali Rizvi, Fatima Safi Arslan, Russell Seth Martins, Maria Khan, Syed Faisal Mahmood

**Affiliations:** ^1^CITRIC Centre for Clinical Best Practices, Aga Khan University, Karachi, Pakistan; ^2^Department of Medicine, Aga Khan University, Karachi, Pakistan; ^3^Aga Khan University, Karachi, Pakistan

## Abstract

**Background:**

The coronavirus disease 2019 (COVID-19) pandemic has presented as a therapeutic challenge for clinicians worldwide due to its rapid spread along with evolving evidence and understanding of the disease. Internationally, recommendations to guide the management of COVID-19 have been created and updated continuously by the WHO and CDC, which have been locally adapted by different countries. Similarly, Pakistan's National Command Operation Center (NCOC), in its national COVID-19 management strategy, generated guidelines for national implementation. Keeping the guidelines updated has proved challenging globally and locally. Here, we present a summary of the process to assess the evidence, including a time-restricted systematic review based on NCOC Clinical Management Guidelines for COVID-19 Infections v4 published on 11^th^ December 2020 version, correlating it with current recommendations and with input one of the guidelines authors, particularly noting the methodological challenges.

**Methods:**

We conducted a systematic review synthesizing global research on treatment options for COVID-19 hospitalized patients, limiting it to pharmacological interventions for hospitalized COVID-19 patients included in Pakistan's NCOC's national guidelines v4 published on 11^th^ December 2020. Each treatment recommendation's strength and quality of evidence was assessed based on the grading of recommendations assessment, development, and evaluation (GRADE) methodology. These were then compared to the most current living WHO COVID-19 pharmacological treatment guidelines v7.1. One of the authors of the NCOC guidelines reviewed and commented on the findings as well.

**Results:**

We note that the data from our systematic review strongly supports corticosteroids use in treating severe and critically ill COVID-19 hospitalized patients correlating with WHO v7.1 guidelines 24 September 2021. However, evidence from our review and WHO v7.1 for the use of tocilizumab had some conflicting evidence, with data from our review until December 2020 supporting only a weak recommendation for its use, compared to the strong recommendation by the WHO for the use of tocilizumab in patients with severe or critical COVID-19 infection. Regarding the use of antibiotics and ivermectin use in treating COVID-19 hospitalized patients, data from our review and WHO v 7.1 recommend against their use.

**Conclusion:**

Research data about the efficacy and safety of pharmacological interventions to treat hospitalized patients with COVID-19 are rapidly evolving, and based on it, the evidence for or against recommendations changes accordingly. Our study illustrates the challenges of keeping up with the evidence; the recommendations were based on studies up till December 2021, and we have compared our recommendations with the WHO v7.1, which showed some significant changes in the use of pharmacological treatment options.

## 1. Introduction

Pakistan, like other countries worldwide, has seen many cases of coronavirus disease 2019 (COVID-19) since the pandemic began [1]. The national government-led response included the creation of a central National Command Operation Center (NCOC), setting up designated hospitals, isolation testing facilities, and following dedicated treatment guidelines based on WHO recommendations. National guidelines were created, 2nd April 2020 (v1), with most recent version (v4) currently in use, “Clinical Management Guidelines for COVID-19 Infections” published by the Government of Pakistan on 11^th^ December 2020 [2]. These have been formulated by national experts incorporating international guidelines and adapting them to local contexts.

## 2. Methods

Here, we present a summary of the process to assess the evidence, including a time-restricted systematic review based on NCOC version, correlating it with current recommendations specifically looking at it from a local perspective; along with input from NCOC guidelines.

We describe the methodological challenges that exist in the developing evidence-based guidelines for an evolving pandemic. We performed a systematic review to evaluate the interventions noted in the NCOC guidelines v4 and to GRADE recommendations for pharmacological interventions for hospitalized patients. Then, we compared our recommendations to WHO v7.1 COVID-19 therapeutics guidelines and subsequently invited an expert narrative review by NCOC experts specifically looking at it from a local perspective.

This study was conducted at the Center for Clinical Best Practices (CCBP), Clinical and Translational Research Incubator (CITRIC), Aga Khan University, Karachi, Pakistan, after approval from the institutional ethical review committee.

The systematic review was conducted following the Preferred Reporting Items for Systematic Reviews and Meta-Analyses (PRISMA) guidelines [3]. We aimed to review the effectiveness of pharmacological interventions included in the NCOC guidelines on mortality and length of stay in hospitalized patients with COVID-19 including evidence available until 11^th^ December 2020. The WHO clinical progression scale for clinical improvement (ordinal scale) was used to categorize the disease severity for each study. Grading of Recommendations Assessment, Development, and Evaluation (GRADE) criteria were used to evaluate and formulate recommendations for or against and strength by considering the quality of evidence and balance between benefits and harms [4] (Table 1 and Supplementary Figure 1).

Studies in English published from 1^st^ November 2019 to 31^st^ December 2020 were included in the review. Data extraction and synthesis data extraction were performed by two independent investigators using a structured data extraction form to ensure consistency. Any disagreements were noted and resolved by further discussion with a third investigator. The extracted data are given in Tables 2–5). The quality of the final included studies was assessed according to each study design. Observational studies were assessed by the National Institute of Health Study Quality Assessment Tool [5]. Randomized control trials were evaluated by Cochrane Risk of Bias (RoB) [6]. Quasiexperimental studies were assessed by the Cochrane Effective Practice and Organization of Care (EPOC) risk of bias tool [7] (Supplementary Figures 2–9).

## 3. Results

In our systematic review, a total of 122 studies were included (Supplementary Figure 1). Data extracted from these final studies are given in Table 2 (cohort and cross-sectional studies), Table 3 (case-control studies), Table 4 (interventional studies), and Table 5 (quasiexperimental studies).

All drugs in the NCOC v4 guidelines were included, and their efficacy was assessed by evaluating length of hospitalization, mortality, and ordinal scores, and a recommendation was made based on GRADE methodology. As per the NCOC panel recommendation, we additionally included colchicine in the review and comparison of WHO, NCOC v4, and our review between drugs, as given in Table 6.

### 3.1. Corticosteroids

Multiple studies have evaluated the efficacy of corticosteroids in the management of COVID-19 (Tables 2 and 5). We gave strong recommendation for the use of corticosteroids because the studies were showing early recovery in severe and critical patients; however, we gave weak recommendation for its use in noncritical patients as it did not show any positive outcomes. Most of the studies in our systematic review were observational; hence, we gave a moderate quality of evidence.

As per our systematic review, we recommend

- For the use of corticosteroids in severe and critical patients, hospitalized COVID-19 patients. Strong recommendation, moderate-quality evidence.
- Against the use of corticosteroids in nonsevere patients, hospitalized COVID-19 patients. Weak recommendation, moderate-quality evidence.

WHO v7.1 dated 24/9/2021 recommended for the use of systematic corticosteroid rather than no corticosteroids in severe and critical COVID-19 patients.

### 3.2. Tocilizumab

In our systematic review, mortality rates with tocilizumab therapy ranged from 6.98% to 60% (depending on patient inclusion criteria), with many studies showing a protective effect of tocilizumab with regards to mortality, especially if given intravenously (as compared to subcutaneously) and within 12 days of admission [8–28]. Patients treated with tocilizumab alone were more likely to show improvement on the WHO ordinal scale (63.9% vs. 36.1%) and less likely to require ICU care (40.4% vs. 59.6%) as compared to those treated with corticosteroids in addition to tocilizumab [29]. We gave weak recommendation as the studies were showing controversial results on managing COVID-19 hospitalized adults with tocilizumab. Many of the studies were showing inconsistent results; therefore, we gave moderate certainty.

As per our systematic review, we recommend

- For the use of tocilizumab in hospitalized COVID-19 patients. Weak recommendation, moderate-quality evidence.

WHO v7.1 recommended for the use of tocilizumab in patients with severe or critical COVID-19 infection.

### 3.3. Ivermectin Therapy

Due to its effectiveness in various other viral infections, ivermectin was assessed as a therapeutic agent for COVID-19 infection (Tables 2 and 4). As studies showed no major difference in mortality, we gave weak recommendation for the use of ivermectin and low quality of evidence because of insufficient evidence.

As per our systematic review, we recommend

- Against the use of ivermectin therapy in the use of COVID-19 hospitalized patients. Weak recommendation, low-quality evidence.

WHO v7.1 recommended against the use of ivermectin in patients with COVID-19.

### 3.4. Antibiotics

The macrolide azithromycin has demonstrated antiviral activity, especially in human bronchial epithelial cells where it reduces viral cell replication and causes an increase in viral-induced pattern recognition receptors. It has exhibited a synergistic effect with the drug hydroxychloroquine, and together, they decrease the production of inflammatory cytokines such as IL-1 and IL-6 [30]. We gave weak recommendation for the management of COVID-19 hospitalized adults with hydroxychloroquine alone or in combination with other antibiotics because the studies were not showing positive outcomes on mortality and length of stay. We gave moderate certainty of evidence as most of the studies in our systematic review were cohort and case-control studies.

As per our systematic review, we recommend

- Against the use of antibiotics, including hydroxychloroquine alone or in combination with other antibiotics in hospitalized COVID-19 patients. Weak recommendation, moderate-quality evidence.

WHO v7.1 recommended against the use of hydroxychloroquine or chloroquine for treatment of COVID-19.

### 3.5. Anticoagulation Therapy

As per our systematic review, the therapeutic doses of anticoagulation, i.e., nadroparin calcium (2850 IU) reduced mortality as compared to the prophylactic doses in the majority of studies [31–34]. While few studies found no difference, Billet et al. reported mortality reduction for prophylactic doses of both apixaban and enoxaparin and therapeutic doses of apixaban but not enoxaparin [35–38]. Therapeutic doses of apixaban did not provide an additional mortality reduction compared to prophylactic doses. Therapeutic doses of anticoagulation were shown to reduce the incidence of a venous thromboembolic event; however, both therapeutic and prophylactic doses of anticoagulation reduce in-hospital mortality compared to patients not receiving anticoagulation [31, 33, 35, 39]. We gave strong recommendation to both therapeutic and prophylactic doses of anticoagulants, as in several studies, it has shown to reduce mortality. Many studies in our systematic review were observational, so we rated the evidence as moderate.

As per our systematic review, we recommend

- For the use of anticoagulant therapeutic doses. Therapeutic: strong recommendation, moderate-quality evidence.
- For the use of prophylactic dose anticoagulants to treat COVID-19 hospitalized patients. Prophylactic: strong recommendation, moderate-quality evidence.

No evidence available as per WHO v7.1.

### 3.6. Antivirals

Remdesivir, an antiviral agent, has been associated with lower mortality, with one study reporting 62% lower odds of mortality and greater clinical improvement [40, 41]. However, the results of other studies have not been as conclusive. Studies using the WHO ordinal scale have found weak associations between remdesivir use and improved patient outcomes [42]. With regards to other antivirals, there are conflicting data from studies. Varying WHO ordinal scale results were found for antivirals lopinavir-ritonavir. Multiple studies found no difference in mortality with the combination of ritonavir/lopinavir and remdesivir [43, 44]. We gave weak recommendation for the use of remdesivir because of the inconsistent results of the studies. However, we recommend against the use of ritonavir/lopinavir use due to conflicting research evidence. Most of the studies on remdesivir were randomized controlled trials; therefore, we rated it as high quality of evidence, while studies on ritonavir/lopinavir were mostly observational, so it has moderate certainty.

As per our systematic review, we recommend

- For the use of remdesivir in hospitalized COVID-19 patients. Weak recommendation, high-quality evidence.
- Against the use of ritonavir/lopinavir in hospitalized COVID-19 patients. No recommendation, moderate-quality evidence.

WHO v7.1: conditional recommendation against administering remdesivir in addition to usual care.

WHO v7.1 recommended against administering lopinavir/ritonavir for treatment of COVID-19.

### 3.7. Convalescent Plasma

Convalescent plasma initially was looked at as a possible therapy for COVID-19 infection due to its prior usefulness in other epidemic viruses. Some initial observational studies suggested the use of convalescent plasma in improving pulmonary function, decreasing adverse effects, increasing survival, and shortening hospital stay [45–47]. While most observational studies reported positive outcomes, RCTs have not supported convalescent plasma use. We gave weak recommendation for convalescent plasma use because RCTs have not shown significant improvement in patients' health status. As many of the studies were observational, therefore, we gave it a moderate quality of evidence.

As per our systematic review, we recommend

- Against the use of convalescent plasma in the management of hospitalized COVID-19 patients. Weak recommendation, moderate-quality evidence.

There are no available evidence as per WHO v7.1.

### 3.8. Famotidine

While famotidine is conventionally used as an H2 receptor blocker, it also has antiviral properties [48]. Freedberg et al., in a single-centered retrospective cohort study, reported reduced mortality with famotidine use [49]. Similarly, when comparing a treatment regimen of HCQ, azathioprine, remdesivir, and corticosteroids with famotidine and those without famotidine, Mather et al. reported lower mortality in the famotidine arm (14% in the famotidine group vs. 26% in the nonfamotidine group) [50]. Despite these results, larger studies and RCTs have not yet established the role of famotidine, and therefore, we gave it low certainty. Given the minimal side effect profile of famotidine, our judgement based on data available at the time was for its use but with weak recommendation.

As per our systematic review, we recommend

- For the use of famotidine in hospitalized COVID-19 patients. Weak recommendation, low-quality evidence.

There are no available evidence as per WHO v7.1.

### 3.9. Immunoglobulin Therapy

Based on the limited number of studies, a retrospective cohort study conducted by Shao et al. found IVIG therapy to reduce the 28-day mortality in critically ill patients (27% in the IVIG group vs. 53% in the non-IVIG group) [51]. Similarly, a randomized trial by Gharebaghi et al. also proved that IVIGs reduced mortality [52]. Studies have shown lower mortality; therefore, we supported its use with weak recommendation. As no significant interventional studies are supporting its use, we gave it moderate certainty.

As per our systematic review, we recommend

- For the use of immunoglobulin therapy in hospitalized COVID-19 patients. Weak recommendation, moderate-quality evidence.

There are no available evidence as per WHO v7.1.

### 3.10. Colchicine

Colchicine works by inhibiting the assembly of microtubules during mitosis by binding to tubulin inside cells and forming tight tubulin-colchicine complexes. This is its major anti-inflammatory mechanism of action [53]. In our review, only 1 single-center cohort of adult hospitalized patients with COVID-19 pneumonia and acute respiratory distress syndrome was found using colchicine 1 mg/day, investigating the association between colchicine use and improved survival in adult hospitalized patients with COVID-19 pneumonia and acute respiratory distress syndrome and found a 20.8% decrease in mortality amongst patients treated with colchicine along with other standards of care drugs [54]. Because of the insufficient research evidence, we are not recommending colchicine use and gave it a low quality of evidence.

As per our systematic review, we recommend

- Against the use of colchicine in hospitalized COVID-19 patients. No recommendation, low-quality evidence.

There are no available evidence as per the WHO v7.1.

## 4. Discussion

A great breadth of literature exists on the therapeutic management of hospitalized adults with COVID-19 based on disease severity, much of which have been analyzed and appraised systematically [55]. Currently, living systematic reviews provide information to guide clinical practice [56, 57]. However, this approach assumes that all treatment options are available or approved in each country or region, which is not the case. In Pakistan, the Government of Pakistan has released Clinical Management Guidelines for COVID-19 Infections, based on which clinicians are expected to prescribe and treat patients [2]. While such guidelines are beneficial in which they are region-specific and keep in mind various economic factors and drug availability, they are not regularly updated due to the resources required for systematic reviews and evidence synthesis. To consolidate the pool of information to assess the effectiveness of currently approved therapeutic options for COVID-19 infection in Pakistan, we have synthesized data relating to only those treatments that are recommended in our country's guidelines using global data from 1^st^ November 2019 to December 31^st^, 2020 [2].

Of all the drug classes analyzed, corticosteroids were found to have the most consistent effect on mortality and length of stay. The WHO clinical progression (ordinal) scale showed a reduction in mortality among patients being treated with corticosteroids. All but a few studies support the use of corticosteroids in patients hospitalized with COVID-19 [58, 59]. Trials that assessed anticoagulation (especially therapeutic vs. prophylactic dosages) were also predominantly found to improve thromboembolic outcomes and mortality. However, variations in the type of anticoagulant used make it hard to recommend a single drug. Nadroparin, tinzaparin, dalteparin (LMWH), heparin, apixaban, and enoxaparin were all found to have reduced mortality [31, 33, 38]. Trials assessing tocilizumab supported its use to limit mortality and length of stay [13, 28, 29, 60]. One study found that the addition of corticosteroids to tocilizumab was a significant protective factor against mortality [61].

Studies assessing remdesivir failed to show any conclusive difference in mortality and length of stays of patients with COVID-19 [42]. Of the antivirals that are being used for COVID-19, lopinavir and ritonavir have predominantly been assessed by various observational studies as well as clinical trials, both of which have been uncertain. Ribavirin alone and in combination with other antivirals (lopinavir/ritonavir + interferon-alpha) was also shown to have minimal efficacy [62]. Data from RCTs led to recommendation against the use of convalescent plasma [63–66], which in the early days of COVID-19 was looked at as a major intervention.

Very few studies have been conducted on famotidine (an H2 receptor blocker) and IVIG. Studies have reported a significantly reduced mortality in patients being treated by famotidine or IVIG compared to control groups; however, further randomized trials and data are needed to make a concrete recommendation. Studies assessing ivermectin also report divided results and highlight the need for further studies. One of the studies assessing colchicine has reported better outcomes in adult hospitalized patients with COVID-19 pneumonia [30].

Our review is the first one to systematically review the drugs specified by the Government of Pakistan's Clinical Management Guidelines for COVID-19 Infections v4, and several other systematic reviews have been conducted to assess the efficacy of drugs used in the treatment of COVID-19, with the WHO living guidelines for pharmacological management including the most up to date data. It was thus imperative that our findings be compared and assessed against broader literature that has been published.

Our review found the lack of randomized trials to be a limitation for evidence regarding less extensively studied agents, while the more extensively investigated agents had been studied by randomized trials. Since then, newer studies, that have been conducted and included in our review, have shown tocilizumab, IVIGs, and colchicine to be effective as well. Larger trials, such as the solidarity trials, have since proven that remdesivir is not effective [67].

The urgency of information about COVID-19 infection treatment has resulted in poorly organized studies that use a variety of different outcome measures, which deters meaningful comparison between different therapeutic agents. Indeed, our review reported a wide range of outcome measures, resulting in difficulty synthesizing data.

To standardize outcome measures across studies, several international bodies worked in union and formulated a set of outcome measures, which included the WHO clinical progression scale. This is an ordinal scale, ranging from 0 (no infection) to 10 (mortality) that is especially useful in widespread diseases. The lower scores (for mild disease, which may or may not require assistance) are more subjective, and the higher scores (of severe disease requiring different levels of intervention) are likely to change based on regional practices. However, the scale is quick to use because the data required are readily available in medical records. Despite its usefulness in standardizing clinical research, the uptake of this scale has not been encouraging [68]. Our systematic review reports only 13 studies that have used this scale to report clinical progression. Gaps in reporting, with different studies grouping or failing to mention the number of patients for each score, undermines the use of a standardized scale to make sound accurate comparisons of clinical data. We recommend the use of the WHO clinical progression scale as a standard practice for studies on COVID-19 infection, with full reporting of all scores to enable comparison of study outcome measures and optimize the systematic analysis of clinical data.

There are several limitations to this systematic review, mostly stemming from considerable heterogeneity between articles. These include variations in participant inclusion criteria of studies, variations in outcome measures, variations in drugs used across the same class, variations in drug dosages, and variations in geographic locations and patient populations across studies. In addition, the retrospective nature of many studies, the limited sample sizes, and inadequate statistical adjustment for reported associations also adversely impact interpretability.

## 5. Conclusion

Data on pharmacological interventions to treat COVID-19 are rapidly evolving, and based on it, the recommendations have also been changed. In our systematic review, the recommendations were based on studies up till December 2021, and we have compared our recommendations with the WHO v7.1, which showed significant changes in recent treatment modalities of COVID-19 infection. Our understanding regarding the management of COVID-19 has evolved rapidly over the last two years and continues to do so. Given the urgent need to offer any therapeutic option, interim recommendations were often made based on the best available data at the time. These data were, however, often from studies that were exploratory or not as rigorously done. This is apparent in the disparate recommendation between the 2 guidelines and the systematic review (which is only looking at studies published during the early part of the pandemic). This also brings to light the need to continually assess the literature and be able to ready to change (previously established) therapeutic recommendations.

## Figures and Tables

**Table 1:** Selection criteria and search strategy.

Characteristics	Inclusion criteria	Exclusion criteria	Search string
Study participants	Studies including adult human participants/patients (age ≥18 years) of either sex with a confirmed diagnosis of COVID-19 in a hospital setting.	Studies including pregnant women.	(COVID-19 [MeSH] OR corona^*∗*^ OR SARS-CoV-2 OR “coronavirus disease” OR “coronavirus infection”[MeSH] OR “Severe acute respiratory syndrome coronavirus 2” OR “coronavirus-2019” OR “novel coronavirus” OR “COVID-19 pandemic” OR 2019nCoV) AND (adult^*∗*^[MeSH] OR young adult^*∗*^[MeSH] OR “adulthood”) AND (“admission” OR “admitted inpatient” OR “in-patient” [MeSH] OR “Hospitalization” [MeSH] OR “Hospitalized” [MeSH] OR “stay”) AND
Interventions	Observational and interventional studies describing the use of the following pharmacologic interventions for the treatment of COVID-19: (i) Steroids (dexamethasone, hydrocortisone, and prednisone methylprednisolone) (ii) Anticoagulation (iii) Remdesivir (iv) Antibiotics (v) Colchicine (vi) Tocilizumab (vii) Other investigational therapies (convalescent plasma, intravenous immunoglobulin, plasmapheresis, ivermectin, and famotidine).	Pharmacologic or nonpharmacologic treatment interventions other than those specified in inclusion criteria.	(intervention^*∗*^ OR drug^*∗*^ OR pharma^*∗*^ OR medic^*∗*^ OR treatment^*∗*^) AND (“Hydroxychloroquine”[Mesh] OR “Azithromycin”[Mesh] OR “Doxycycline”[Mesh] OR “Amoxicillin”[Mesh] OR anticoagul^*∗*^ OR “ Low-Molecular-Weight Heparin” OR “Unfractionated Heparin” OR “remdesivir”[MeSH] OR “antibiotics”[MeSH] OR “antiviral” OR “investigational therapies”[MeSH] OR “convalescent plasma” OR “intravenous immunoglobulin”[MeSH] OR “plasmapheresis”[MeSH] OR “ivermectin”[MeSH] OR “famotidine”[MeSH] OR “tocilizumab” OR steroid^*∗*^ OR “dexamethasone”[MeSH] OR “hydrocortisone”[MeSH] OR “Methylprednisolone” OR “prednisone”[MeSH]) OR “colchicine”[MeSH]) AND
Outcomes	Studies describing at least one of the following primary or secondary outcome measures: (a) Primary outcomes: (i) In-hospital mortality (ii) Length of hospital stay. (b) Secondary outcomes: (i) Progression of disease (ii) Treatment of adverse effects	—	(“survival” [MeSH] OR recover^*∗*^ OR discharge^*∗*^ OR “death” [MeSH] OR “mortality” [MeSH] OR “fatality”)

**Table 2:** Description of study characteristics: cross-sectional and cohort studies.

S. no.	Author, country	Title	Study duration in days	Intervention group	Comparator group	Concomitant group	Outcome measures	Outcome measures applicable to this review
1	Stessel et al., Belgium	Impact of implementation of an individualized thromboprophylaxis protocol in critically ill ICU patients with COVID-19: A longitudinal controlled before-after study.	52	Nadroparin calcium 2850 IU (preimplementation of protocol)	Nadroparin calcium 2850 IU (postimplementation of protocol)	—	(i) One-month mortality (ii) Two-week and three-week mortality (iii) Hospital length of stay	(i) One-month mortality (ii) Two-week and three-week mortality (iii) Hospital length of stay
2	Jonmarker et al., Sweden	Dosing of thromboprophylaxis and mortality in critically ill COVID-19 patients	56	(i) Tinzaparin or dalteparin low (2500–4500 IU tinzaparin or 2500–5000 IU dalteparin) (ii) Tinzaparin or dalteparin medium (> 4500 IU but < 175 IU/kilogram, kg, of bodyweight tinzaparin or > 5000 IU but < 200 IU/kg of bodyweight dalteparin) (iii) High dose (≥ 175 IU/kg of bodyweight tinzaparin or ≥ 200 IU/kg of bodyweight dalteparin)	—	—	(i) 28 days mortality (ii) ICU stay (iii) Thromboembolic events	(i) 28 days mortality (ii) ICU stay (iii) Thromboembolic events
3	Salton et al., Italy	Prolonged low-dose methylprednisolone in patients with severe COVID-19 pneumonia	58	Methylprednisolone loading dose of 80 mg intravenously, followed by an infusion of 80 mg/d in 240 ml of normal saline at 10 ml/h for at least 8 days	—	Standard of care (antibiotics, antivirals, vasopressors, and renal replacement therapy)	(i) Mortality (ii) Transfer to intensive care unit (iii) Invasive mechanical ventilation	(i) Mortality (ii) Transfer to intensive care unit (iii) Invasive mechanical ventilation
4	Mutair et al., Saudi Arabia	Clinical, epidemiological, and laboratory characteristics of mild-to-moderate COVID-19 patients in Saudi Arabia: An observational cohort study	31	Hydroxychloroquine in mild cases	Hydroxychloroquine in moderate cases	(i) Hydroxychloroquine (ii) Azithromycin (i) Oseltamivir (iii) Vitamin C (iv) Vitamin E (v) Ceftriaxone (vi) Enoxaparin	(i) Days of hospitalization (ii) SARS-CoV-2 PCR negative (iii) Treatment outcomes	(i) Days of hospitalization (ii) SARS-CoV-2 PCR negative (iii) Treatment outcomes
5	Annie et al., the United States	Hydroxychloroquine in hospitalized patients with COVID-19: real-world experience assessing mortality	116	(i) Hydroxychloroquine alone (ii) Hydroxychloroquine plus azithromycin	—	—	Mortality	Mortality
6	Arshad et al., the United States	Treatment with hydroxychloroquine, azithromycin, and combination in patients hospitalized with COVID-19	—	(i) Hydroxychloroquine alone (ii) Azithromycin alone 500 mg once daily on day 1 followed by 250 mg once daily for the next 4 days. (iii) Hydroxychloroquine plus azithromycin	Neither treatment	(i) Steroid (ii) Tocilizumab	(i) Mortality (ii) Hospital length of stay in days.	(i) Mortality (ii) Hospital length of stay in days
7	Ashinyo et al., Ghana	Clinical characteristics, treatment regimen, and duration of hospitalization among COVID-19 patients in Ghana: A retrospective cohort study	93	(i) Chloroquine + hydroxychloroquine (ii) Hydroxychloroquine + azithromycin (iii) Hydroxychloroquine only (iv) Azithromycin only (v) Supportive treatment	—	—	Duration of hospitalization	Duration of hospitalization
8	Ayerbe et al., Spain	The association between treatment with heparin and survival in patients with COVID-19	55	(i) Heparin	—	(i) Hydroxychloroquine (ii) Azithromycin (iii) Steroids (iv) Tocilizumab (v) Lopinavir with ritonavir (vi) Oseltamivir	Mortality	Mortality
9	Ayerbe et al., Spain	The association of treatment with hydroxychloroquine and hospital mortality in COVID-19 patients	55	Hydroxychloroquine was dosed as 400 mg twice daily the first day, followed by 200 mg twice daily for 4–6 days.	—	(i) Azithromycin (ii) Steroids (iii) Heparin (iv) Tocilizumab (v) Lopinavir with ritonavir (vi) Oseltamivir	Mortality	Mortality
10	Bartoletti et al., Italy	Efficacy of corticosteroid treatment for hospitalized patients with severe COVID-19: A multicentre study	130	Corticosteroid ≥0.5 mg/kg of prednisone	—	(i) Hydroxychloroquine (ii) Lopinavir/ritonavir (iii) Darunavir/ritonavir (iv) Darunavir/cobicistat (v) Remdesivir (vi) Low-molecular-weight heparin (vii) Other standard of care	Mortality	Mortality
11	Camprubí et al., Spain	Lack of efficacy of standard doses of ivermectin in severe COVID-19 patients	21	Ivermectin 200 *μ*g/kg for 8–18 days	No ivermectin	(i) Tocilizumab (ii) Steroids (iii) Anakinra (iv) Remdesivir (v) Lopinavir/ritonavir (vi) Hydroxychloroquine (vii) Azithromycin (viii) Beta-interferon (ix) Hydroxychloroquine (x) Azithromycin	Severe adverse events	Severe adverse events
12	Canoglu et al., Turkey	Therapeutic dosing of low-molecular-weight heparin may decrease mortality in patients with severe COVID-19 infection	51	Heparin prophylactic dose LMWH (0.5 mg/kg twice daily)	Heparin therapeutic dose LMWH (1 mg/kg twice daily)	(i) Favipiravir (ii) Lopinavir-ritonavir (iii) Supportive treatment	(i) Mortality (ii) ICU admission (iii) Hospital stay	(i) Mortality (ii) ICU admission (iii) Hospital stay
13	Catteau et al., Belgium	Low-dose hydroxychloroquine therapy and mortality in hospitalized patients with COVID-19: A nationwide observational study of 8075 participants	72	Hydroxychloroquine 2400 mg over 5 days	No hydroxychloroquine	(i) Lopinavir/ritonavir (ii) Hydroxychloroquine (iii) Tocilizumab (iv) Remdesivir (v) Macrolides (vi) Anakinra	(i) Mortality (ii) Hospital stay (iii) Invasive mechanical ventilation (iv) Admission to ICU	(i) Mortality (ii) Hospital stay (iii) Invasive mechanical ventilation (iv) Admission to ICU
14	Ana Fernández-Cruz, Spain	A retrospective controlled cohort study of the impact of glucocorticoid treatment in SARS-CoV-2 infection mortality	35	Steroid	No steroid	(i) Hydroxychloroquine (ii) Lopinavir-ritonavir (iii) Azithromycin (iv) Interferon (v) Tocilizumab (vi) Anakinra	Mortality	Mortality
15	Freedberg et al., the United States	Famotidine use is associated with improved clinical outcomes in hospitalized COVID-19 patients: A propensity score matched retrospective cohort study	56	Famotidine	No famotidine	—	Mortality or intubation	Mortality or intubation
16	Geleris et al., United States	Observational study of hydroxychloroquine in hospitalized patients with COVID-19	50	Hydroxychloroquine 600 mg twice on day 1 and then 400 mg daily for a median of 5 days	No hydroxychloroquine	(i) Systemic glucocorticoid (ii) Anticoagulant or warfarin (iii) Azithromycin (iv) Antibiotic agent (v) Tocilizumab (vi) Remdesivir	Intubation or death	Intubation or death
17	Berry et al., United States	Hydroxychloroquine and tocilizumab therapy in COVID-19 patients: An observational study	66	(i) Hydroxychloroquine 800 mg on day 1 and 400 mg on days 2–5, followed by 200 mg TID (ii) Hydroxychloroquine in combination with azithromycin (iii) Tocilizumab first dose 400 mg, followed by 800 mg	(i) Neither hydroxychloroquine/azithromycin (ii) Azithromycin alone (iii) No tocilizumab	For patients in tocilizumab/no tocilizumab group: (i) Steroid (ii) Hydroxychloroquine alone (iii) Azithromycin alone (iv) Azithromycin plus hydroxychloroquine	(i) Mortality (ii) Adverse drug events	(i) Mortality (ii) Adverse drug events
18	Karolyi et al., Austria	Hydroxychloroquine versus lopinavir/ritonavir in severe COVID-19 patients	57	Hydroxychloroquine loading dose of 400 mg twice on the first day, followed by 200 mg twice daily	Lopinavir/ritonavir 400 mg/100 mg administered twice daily	Concomitant antibiotic	(i) In-hospital mortality (ii) Intensive care unit (ICU) admission (iii) Length of stay (iv) PCR (polymerase chain reaction) negativity (v) Side effects of treatment	(i) In-hospital mortality (ii) Intensive care unit (ICU) admission (iii) Length of stay (iv) PCR (polymerase chain reaction) negativity (v) Side effects of treatment
19	Kirenga et al., Uganda	Characteristics and outcomes of admitted patients infected with SARS-CoV-2 in Uganda	—	Hydroxychloroquine	No hydroxychloroquine	(i) Antibiotics (azithromycin, ampicillin/cloxacillin combination, and augmentin) (ii) Vitamin C	(i) Admission to ICU (ii) Mechanical ventilation (iii) Death (iv) Negative reverse transcriptase PCR (RT-PCR) tests (v) Length of hospitalization	(i) Admission to ICU (ii) Mechanical ventilation (iii) Death (iv) Negative reverse transcriptase PCR (RT-PCR) tests (v) Length of hospitalization
20	Lagier et al., France	Outcomes of 3737 COVID-19 patients treated with hydroxychloroquine/azithromycin and other regimens in Marseille, France: A retrospective analysis	56	(i) Azithromycin + hydroxychloroquine >3 days (hydroxychloroquine 200 mg of oral hydroxychloroquine, 3 times daily for 10 days and 500 mg of oral azithromycin on day 1 followed by 250 mg daily for the next 4 days) (ii) Other treatment (azithromycin-hydroxychloroquine for at least 3 days) (iii) Azithromycin + hydroxychloroquine <3 days (iv) Hydroxychloroquine alone (v) Azithromycin alone (vi) No azithromycin and hydroxychloroquine	—	—	(i) Death (ii) Transfer to the intensive care unit (ICU) (iii) ≥10 days of hospitalization (iv) Viral shedding	(i) Death (ii) Transfer to the intensive care unit (ICU) (iii) ≥10 days of hospitalization (iv) Viral shedding
21	Albertini et al., France	Observational study on off-label use of tocilizumab in patients with severe COVID-19	16	Tocilizumab 8 mg/kg	No tocilizumab	(i) Hydroxychloroquine (ii) Azithromycin	(i) Mortality (ii) Mechanical ventilation (iii) Length of stay (iv) Adverse events	(i) Mortality (ii) Mechanical ventilation (iii) Length of stay (iv) Adverse events
22	Almazrou et al., Saudi Arabia	Comparing the impact of hydroxychloroquine-based regimens and standard treatment on COVID-19 patient outcomes: A retrospective cohort study	20	Hydroxychloroquine	Standard care (i) Oseltamivir (ii) Azithromycin (iii) Levofloxacin (iv) Hydroxychloroquine (v) Ceftriaxone (vi) Piperacillin/tazobactam (vii) Vancomycin (viii) Cefuroxime (ix) Doxycycline (x) Tazocin (xi) Moxifloxacin	—	(i) Hospital length of stay (ii) Time in ICU, days (iii) ICU admission (iv) Mechanical ventilation	(i) Hospital length of stay (ii) Time in ICU, days (iii) ICU admission (iv) Mechanical ventilation
23	Billet et al., the United States	Anticoagulation in COVID-19: effect of enoxaparin, heparin, and apixaban on mortality	30	(i) Apixaban prophylaxis (ii) Apixaban full therapy (iii) Enoxaparin prophylaxis (iv) Enoxaparin full therapy (v) Unfractionated Heparin standard prophylaxis (vi) Unfractionated heparin high prophylaxis (vii) Unfractionated heparin full therapy	—	—	(i) Mortality (ii) Respiratory support	(i) Mortality (ii) Respiratory support
24	Capra et al., Italy	Impact of low-dose tocilizumab on mortality rate in patients with COVID-19 related pneumonia	—	Tocilizumab	No tocilizumab	Standard care (i) Hydroxychloroquine 400 mg (ii) Lopinavir 800 mg (iii) Ritonavir 200 mg	Mortality	Mortality
25	Mario Fernández‐Ruiz et al., Spain	Tocilizumab for the treatment of adult patients with severe COVID-19 pneumonia: A single-center cohort study	—	Tocilizumab	—	(i) Hydroxychloroquine (ii) Lopinavir (iii) Ritonavir (iv) Azithromycin (v) Interferon (vi) Corticosteroids	(i) Clinical improvement at day 7 (ii) Clinical improvement at day 14	(i) Clinical improvement at day 7 (ii) Clinical improvement at day 14
26	Gupta et al., the United States	Association between early treatment with tocilizumab and mortality among critically ill patients with COVID-19	122	Tocilizumab patients received tocilizumab within 2 days of ICU admission	Tocilizumab patients did not receive tocilizumab within 2 days of ICU admission	(i) Hydroxychloroquine (ii) Azithromycin (iii) Corticosteroid (iv) Anticoagulation	(i) Mortality (ii) Adverse event	(i) Mortality (ii) Adverse event
27	Kaminski et al., the United States	Tocilizumab therapy for COVID-19: A comparison of subcutaneous and intravenous therapies	54	Tocilizumab, 400 mg IV	Tocilizumab, subcutaneous dose of 324 mg (given as two simultaneous doses of 162 mg)	(i) Hydroxychloroquine 400 mg twice for one day, followed by 200 mg twice a day for additional 4 days plus azithromycin 500 mg once, followed by 250 mg oral once daily for additional 4 days. (ii) Tocilizumab therapy (iii) Corticosteroids for 3–5 days	(i) Survival rate (ii) Ventilatory status	(i) Survival rate (ii) Ventilatory status
28	Kim et al., Korea	Lopinavir-ritonavir versus hydroxychloroquine for viral clearance and clinical improvement in patients with mild-to-moderate coronavirus disease 2019	44	Lopinavir-ritonavir 400 and 100 mg twice daily	Hydroxychloroquine 400 mg once daily	(i) Antibiotic agent (ii) Glucocorticoid (iii) IV immunoglobulin	(i) Time to negative conversion of viral RNA (ii) Time to clinical improvement (iii) Adverse events	(i) Time to negative conversion of viral RNA (ii) Time to clinical improvement (iii) Adverse events
29	Lammers et al., Netherlands	Early hydroxychloroquine but not chloroquine use reduces ICU admission in COVID-19 patients	—	Hydroxychloroquine on day 1, 400 mg, and 400 mg after 12 hours, 200 mg BID on days 2–5	Chloroquine on 1st day 600 mg and 300 mg after 12 h, 300 mg BID on days 2–5	Azithromycin	(i) Death (ii) Transfer to the intensive care unit (ICU)	(i) Death (ii) Transfer to the intensive care unit (ICU)
30	Lauriola et al., Italy	Effect of combination therapy of hydroxychloroquine and azithromycin on mortality in patients with COVID-19	—	(i) Azithromycin and hydroxychloroquine (hydroxychloroquine dose of 200 mg TID (alone or in combination) and azithromycin 500 mg QD for 10 days) (ii) Hydroxychloroquine 200 mg TID	No treatment (standard care not specified)	—	Mortality	Mortality
31	Lee et al., the United States	Remdesivir for the treatment of severe COVID-19: A community hospital's experience	111	Remdesivir 200 mg loading dose on day 1, followed by a 100 mg daily on days 2–5	—	(i) Antibiotics (ii) Convalescent plasma (iii) Dexamethasone	(i) Mortality (ii) Length of stay (iii) ICU admission	(i) Mortality (ii) Length of stay (iii) ICU admission
32	Yiming Li et al., China	Corticosteroid therapy in critically ill patients with COVID-19: A multicenter, retrospective study	62	Corticosteroids	No corticosteroids	—	(i) 90 days mortality (ii) Viral clearance	(i) 90 days mortality (ii) Viral clearance
33	Liu et al., China	Clinical characteristics and corticosteroids application of different clinical types in patients with coronavirus disease 2019	121	(i) Corticosteroid (ii) Methylprednisolone (1-2) mg/kg day general type, 1–5 mg/kg day severe type, and 1–4 mg/kg day critical type	No corticosteroids	(i) Interferon-*α* (IFN-*α*) (ii) Lopinavir/ritonavir	(i) Discharges (ii) Mechanical ventilation (iii) Intensive care unit (ICU) admission (iv) Mortality	(i) Discharges (ii) Mechanical ventilation (iii) Intensive care unit (ICU) admission (iv) Mortality
34	Gautret et al., France	Clinical and microbiological effect of a combination of hydroxychloroquine and azithromycin in 80 COVID-19 patients with at least a 6-day follow-up: A pilot observational study	—	Hydroxychloroquine (200 mg of oral TID for 10 days) and azithromycin (500 mg on day 1 followed by 250 mg per day for 4 days)	—	—	(i) Requiring oxygen therapy or transfer to the ICU after at least three days of treatment (ii) Length of stay in the infectious diseases ward (iii) Contagiousness as assessed by PCR and culture	(i) Requiring oxygen therapy or transfer to the ICU after at least three days of treatment (ii) Length of stay in the infectious diseases ward (iii) Contagiousness as assessed by PCR and culture
35	Yu et al., China	Low dose of hydroxychloroquine reduces fatality of critically ill patients with COVID-19	—	Hydroxychloroquine oral 200 mg BID for 7–10 days	Nonhydroxychloroquine	(i) Lopinavir and ritonavir (ii) Ribavirin (iii) Intravenous immunoglobulin	(i) Mortality (ii) Length of stay	(i) Mortality (ii) Length of stay
36	Guaraldi et al., Italy	Tocilizumab in patients with severe COVID-19: A retrospective cohort study	—	(i) Tocilizumab 8 mg/kg IV (up to a maximum of 800 mg) in two infusions, 12 h apart, or subcutaneously at 162 mg administered in two simultaneous doses, one in each thigh (i.e., 324 mg in total) (ii) Hydroxychloroquine (iii) Azithromycin (iv) Antiretrovirals (v) Low-molecular-weight heparin	(i) Hydroxychloroquine (ii) Azithromycin (iii) Antiretrovirals (iv) Low-molecular-weight heparin (v) Lopinavir-ritonavir	(i) Hydroxychloroquine (ii) Azithromycin (iii) Antiretrovirals (iv) Low-molecular-weight heparin (v) Lopinavir-ritonavir	(i) Death (ii) Invasive mechanical ventilation	(i) Death (ii) Invasive mechanical ventilation
37	Grein et al., multicenter	Compassionate use of remdesivir for patients with severe COVID-19	—	Remdesivir loading dose of 200 mg intravenously on day 1 plus 100 mg daily for the following 9 days	—	Supportive care	(i) Clinical improvement (ii) Changes in oxygen support requirements (iii) Adverse events (iv) Death	(i) Clinical improvement (ii) Changes in oxygen support requirements (iii) Adverse events (iv) Death
38	Alexis K. Okoh et al., the United States	Tocilizumab use in COVID-19 associated pneumonia	61	Tocilizumab 8 mg/kg IV (maximum: 800 mg/dose)	No tocilizumab	Standard of care	(i) Deescalation in oxygen therapy (ii) In-hospital death (iii) Septic shock (iv) Acute kidney injury (AKI) requiring hemodialysis	(i) Deescalation in oxygen therapy (ii) In-hospital death (iii) Acute kidney injury (AKI) requiring hemodialysis
39	Shao et al., China	Clinical efficacy of intravenous immunoglobulin therapy in critical ill patients with COVID-19: A multicenter retrospective cohort study	122	IVIG 0.1–0.5 g/kg per day	Non-IVIG	—	(i) 28 days mortality (ii) 60 days mortality (iii) In‐hospital days (iv) Total course of disease	(i) 28 days mortality (ii) 60 days mortality (iii) In‐hospital days
40	Kewan et al., the United States	Tocilizumab for treatment of patients with severe COVID-19: A retrospective cohort study	61	Tocilizumab 8 mg/kg and received 400 mg tocilizumab as a 60 min single intravenous infusion	No tocilizumab	(i) Hydroxychloroquine with a loading dose of 400 mg twice daily followed by 200 mg BID for 5 days (ii) Azithromycin 500 mg per day for 5 days (iii) Steroid	(i) Intubated patients' improvement in oxygen support (ii) Noninvasive oxygen support improvement in oxygen support (iii) Clinical improvement among patients required mechanical ventilation (iv) Length of stay in hospital (v) Mortality	(i) Intubated patients' improvement in oxygen support (ii) Noninvasive oxygen support improvement in oxygen support (iii) Clinical improvement among patients required mechanical ventilation (iv) Length of stay in hospital (v) Mortality
41	Ramiro et al., Netherlands	Historically controlled comparison of glucocorticoids with or without tocilizumab versus supportive care only in patients with COVID-19 associated cytokine storm syndrome: results of the CHIC study	92	(i) Methylprednisolone 250 mg intravenously on day 1, followed by MP 80 mg intravenously on days 2–5 (ii) Tocilizumab single dose 8mg/kg bodyweight intravenous, maximum 800 mg between day 2 and day 5	—	(i) Ceftriaxone 2 g every 24 hours for 7 days (ii) Chloroquine 300 mg every 12 hours following a loading dose of 600 mg	(i) Clinical improvement (ii) WHO ordinal scale (iii) Hospital mortality (iv) Mechanical ventilation (v) Duration of hospitalization	(i) Clinical improvement (ii) WHO ordinal scale (iii) Hospital mortality (iv) Mechanical ventilation (v) Duration of hospitalization
42	Colaneri et al., Italy	Tocilizumab for treatment of severe COVID-19 patients: preliminary results from SMAtteo COVID-19 Registry (SMACORE)	14	Tocilizumab 8 mg/kg (up to a maximum 800 mg per dose) IV repeated 12 hours plus standard of care	Standard of care (i) Hydroxychloroquine 200 mg BID (ii) Azithromycin 500 mg once (iii) Prophylactic dose of low weight heparin (iv) Methylprednisolone (a tapered dose of 1 mg/kg up to a maximum of 80 mg) for 10 days	—	(i) ICU admission (ii) 7 days mortality rate (iii) Clinical and laboratory data (iv) Days of hospitalization	(i) ICU admission (ii) 7 days mortality rate (iii) Days of hospitalization
43	Mather et al., the United States	Impact of famotidine use on clinical outcomes of hospitalized patients with COVID-19	80	(i) Famotidine 80 mg (range 40–160 mg) was received over a median of 4 days (ii) Hydroxychloroquine 600 mg/day	(i) No famotidine	(i) Hydroxychloroquine, 600 mg/day (ii) Azithromycin (iii) Remdesivir (iv) Corticosteroids	(i) Death (ii) Disease severity (iii) Mechanical ventilation	(i) Death (ii) Mechanical ventilation
44	Million et al., France	Early treatment of COVID-19 patients with hydroxychloroquine and azithromycin: A retrospective analysis of 1061 cases in Marseille, France	29	Hydroxychloroquine + azithromycin: a combination of 200 mg of oral HCQ, 3 times daily for 10 days combined with 5 of AZ (500 mg on day 1 followed by 250 mg daily for the next 4 days)	Hydroxychloroquine + azithromycin early treatment, as standard care.	—	(i) Length of hospitalization (ii) Death (iii) Contagiousness as assessed by PCR and culture.	(i) Length of hospitalization (i) Death (iii) Contagiousness as assessed by PCR and culture.
45	Morrisona et al., the United States	Clinical characteristics and predictors of survival in adults with coronavirus disease 2019 receiving tocilizumab	34	(i) Tocilizumab was administered as an 8 mg/kg IV dose using actual bodyweight with a maximum dose of 800 mg. Doses were rounded to 400 mg, 600 mg, or 800 mg. (ii) Corticosteroids (iii) Hydroxychloroquine (iv) Lopinavir/ritonavir with ribavirin (v) Remdesivir	(i) Lopinavir/ritonavir with ribavirin, received as supportive care (ii) Hydroxychloroquine, received as supportive care	—	(i) WHO ordinal scale (ii) 28 day in-hospital survival (iii) Duration of hospitalization	(i) WHO ordinal scale (ii) 28 day in-hospital survival (iii) Duration of hospitalization
46	Pasquini et al., Italy	Effectiveness of remdesivir in patients with COVID-19 under mechanical ventilation in an Italian ICU	21	Remdesivir, first dose of 200 mg IV on day 1 plus 100 mg daily from day 2 on.	No remdesivir	(i) Tocilizumab (ii) Hydroxychloroquine (iii) Lopinavir/ritonavir with ribavirin	Mortality	Mortality
47	Patel et al., India	Safety and efficacy of tocilizumab in the treatment of severe acute respiratory syndrome coronavirus 2 pneumonia: A retrospective cohort study	31	Tocilizumab dosed at 8 mg/kg, up to a maximum dose of 800 mg	—	(i) Hydroxychloroquine. 400 mg/day for 5 days after a loading dose of 400 mg twice a day on 1st day. (ii) Azithromycin 500 mg/day for 5 days. (iii) IV ceftriaxone 1 g per day for 5 days. (iv) Anticoagulation: low-molecular-weight heparin or unfractionated heparin infusion was used if the patient's D-dimer was >1000 ng/ml. (v) Methylprednisolone: some patients also received a single dose of 80 mg IV methylprednisolone before receiving tocilizumab.	(i) Mortality (ii) Admission to the intensive care unit (ICU) with invasive mechanical ventilation or death (iii) Hospital stay	(i) Mortality (ii) Admission to the intensive care unit (ICU) with invasive mechanical ventilation or death (iii) Hospital stay
48	Rahmani et al., Iran	Comparing outcomes of hospitalized patients with moderate and severe COVID-19 following treatment with hydroxychloroquine plus atazanavir/ritonavir	58	Hydroxychloroquine + atazanavir/ritonavir has 400 mg BD on the first day and then 200 mg. 300/100 mg daily was started within 24 h of the hospital admission for all patients	(i) ARB (ii) Metformin (iii) Aspirin (iv) Beta-blockers (v) Insulin (vi) Corticosteroids (vii) Hydroxychloroquine (viii) Immunosuppressants (ix) NSAIDs (x) Methotrexate (xi) ACEI (xii) Azathioprine (xiii) Sulfasalazine	(i) Interferon (ii) Ribavirin (iii) Corticosteroid (iv) IVIG (v) Vitamin C (vi) Antibiotics	(i) 28 days mortality (ii) Hospital stay (iii) ICU stays (iv) Rate of ICU admissions and intubation	(i) 28 days mortality (ii) 56 days mortality (iii) Hospital stay (iv) Rate of ICU admissions and intubation
49	Rodrı'guez-MolineroI et al., Spain	Observational study of azithromycin in hospitalized patients with COVID-19	52	Azithromycin prescribed at a dose of 500 mg on the first day (oral or intravenous), followed by 250 mg daily, until completing 5 days of treatment.	Standard of care	(i) Azithromycin (ii) Tocilizumab (iii) Methylprednisolone (iv) Dexamethasone	(i) Hospital stays (ii) Mortality (iii) Oxygen requirement	(i) Hospital stays (ii) Mortality (iii) Oxygen requirement
50	Rosenberg et al., the United States	Association of treatment with hydroxychloroquine or azithromycin with in-hospital mortality in patients with COVID-19 in New York State	19	(i) Hydroxychloroquine with or without azithromycin (ii) Azithromycin	—	—	(i) In-hospital mortality (ii) Cardiac arrest and abnormal electrocardiographic (ECG) findings (defined as arrhythmia or prolonged QT fraction) (iii) Length of stay	(i) In-hospital mortality (ii) Length of stay
51	Rubio-Rivasa et al., Spain	Beneficial effect of corticosteroids in preventing mortality in patients receiving tocilizumab to treat severe COVID-19 illness	22	Tocilizumab as a single IV infusion at a dose of 400 mg (weight <80 kg) or 600 mg (weight >80 kg) or using methylprednisolone at doses ranging from 0.5 mg/kg/d to 250 mg IV in 3 pulses	—	(i) Hydroxychloroquine (ii) Azithromycin (iii) Lopinavir/ritonavir (iv) Remdesivir (v) Prophylactic anticoagulation therapy (vi) Subcutaneous interferon beta-1b 0.25 mg/48 h.	(i) In-hospital mortality	(i) In-hospital mortality
52	Shi et al., China	Evaluation of antiviral therapies for coronavirus disease 2019 pneumonia in Shanghai, China	19	(i) Arbidol group 200 mg, three times/day (ii) Lopinavir/ritonavir group two tablets, two times/day (iii) Arbidol + lopinavir/ritonavir group (iv) Interferon group 100 000 U/kg, two times/day (v) Interferon + lopinavir/ritonavir group (vi) Interferon + darunavir group one tablet, one time/day.	—	—	(i) Improvements in pulmonary involvement (ii) Length of hospital stay	(i) Pneumonia resolution after treatment (ii) Length of hospital stay
53	Tang et al., China	Anticoagulant treatment is associated with decreased mortality in severe coronavirus disease 2019 patients with coagulopathy	34	Unfractionated heparin or low-molecular-weight heparin (LMWH) for 7 days or longer.	No heparin or less than 7 days	—	28 days mortality	28 days mortality
54	Tong et al., China	Ribavirin therapy for severe COVID-19: a retrospective cohort study	60	Intravenous ribavirin 500 mg every 12 h	—	Corticosteroids	(i) Mortality (ii) Negative conversion time for the SARS-CoV-2 RT-PCR test	(i) Mortality (ii) Negative conversion time for the SARS-CoV-2 RT-PCR test
55	Toniati et al., Italy	Tocilizumab for the treatment of severe COVID-19 pneumonia with hyperinflammatory syndrome and acute respiratory failure: A single-center study of 100 patients in Brescia, Italy	12	(i) Tocilizumab. at a dosage of 8 mg/kg (max 800 mg) by two consecutive intravenous infusions 12 h apart. (ii) Lopinavir + ritonavir 400 mg and 100 mg twice a day. (iii) Remdesivir (iv) Azithromycin (v) Ceftriaxone (vi) Piperacillin/tazobactam (vii) Hydroxychloroquine 400 mg/day (viii) Dexamethasone 20 mg/day	—	—	Improvement in acute respiratory failure	Improvement in acute respiratory failure
56	Tsai et al., China	Successful treatment of 28 patients with coronavirus disease 2019 at a medical center in Taiwan	154	(i) Hydroxychloroquine + azithromycin + ceftriaxone + teicoplanin (ii) Hydroxychloroquine + azithromycin + ceftriaxone (iii) Hydroxychloroquine + azithromycin (iv) Hydroxychloroquine + ceftriaxone (v) Hydroxychloroquine, azithromycin	—	—	None	None
57	Vu et al., Florida	Effects of tocilizumab in COVID-19 patients: A cohort study	19	Tocilizumab 400 mg (30–100 kg) and 600 mg (> 100 kg)	—	(i) Hydroxychloroquine (ii) Methylprednisolone (iii) Intravenous immunoglobulin (iv) Convalescent plasma	(i) WHO ordinal scale (ii) Length of stay (iii) Mortality	(i) WHO ordinal scale (ii) Mortality (iii) Length of stay
58	Wu et al., China	Systemic corticosteroids and mortality in severe and critical COVID-19 patients in Wuhan, China	81	(i) Corticosteroid (ii) Hydrocortisone 5 mg (iii) Methylprednisolone 1 mg (iv) Dexamethasone 0.1875 mg	No corticosteroid	—	(i) Mortality (ii) Hospital stays	(i) Mortality (ii) Hospital stays
59	Yan et al., China	Factors associated with prolonged viral shedding and impact of lopinavir/ritonavir treatment in hospitalized noncritically ill patients with SARS-CoV-2 infection	39	(i) Lopinavir/ritonavir 400 mg and 100 mg, orally twice daily (ii) Corticosteroid therapy (iii) Antibiotics (iv) High-flow nasal cannula oxygen therapy (v) Noninvasive mechanical ventilation (vi) Invasive mechanical ventilation	—	—	(i) Length of stay (ii) Viral shedding days	(i) Length of stay (ii) Viral shedding days
60	Yang et al., China	The role of methylprednisolone on preventing disease progression for hospitalized patients with severe COVID‐19	67	Methylprednisolone 50–80 mg/d	(i) Nonmethylprednisolone (ii) Oseltamivir (iii) Arbidol hydrochloride (iv) Lopinavir/ritonavir (v) Darunavir and/cobicistat	—	(i) Progression to critical illness (ii) Deaths	(i) Progression to critical illness (ii) Deaths
61	You et al., China	The use of methylprednisolone in COVID-19 patients: A propensity score matched retrospective cohort study	60	Methylprednisolone 40 mg once or twice per day within 48 hours of admission for one week.	Nonmethylprednisolone	—	(i) Hospital mortality (ii) Positive nucleic acid test to turn negative (iii) Length of hospital stay (iv) Oxygen requirement	(i) Hospital mortality (ii) Positive nucleic acid test to turn negative (iii) Length of hospital stay (iv) Oxygen requirement
62	Yuan et al., China	Effects of corticosteroid treatment for nonsevere COVID-19 pneumonia: A propensity score-based analysis	37	(i) Corticosteroid (ii) Methylprednisolone	Noncorticosteroid group	(i) Ribavirin (ii) Oseltamivir (iii) Arbidol (iv) Lopinavir/ritonavir (v) Interferon	(i) Progressed to severe cases (ii) Secondary infection (iii) Hospital stays (iv) Duration of viral shedding (v) Fever time	(i) Progressed to severe cases (ii) Hospital stay (iii) Duration of viral shedding
63	Mushtaq et al., Pakistan	Outcome of COVID-19 patients with use of tocilizumab: A single-center experience	62	Tocilizumab 4–8 mg/kg.	—	(i) Azithromycin (ii) Ceftriaxone or piperacillin/tazobactam (iii) Methylprednisolone (iv) Hydroxychloroquine	(i) Mortality (ii) Length of hospital stay (iii) Weaning from a mechanical ventilator, weaning from oxygen support, improvement in laboratory parameters	(i) Mortality (ii) Length of hospital stay (iii) Weaning from a mechanical ventilator, weaning from oxygen support, improvement in laboratory parameters
64	Yan Zuo et al., China	Lopinavir/ritonavir and interferon combination therapy may help shorten the duration of viral shedding in patients with COVID‐19: A retrospective study in two designated hospitals in Anhui, China	56	(i) Corticosteroid (ii) Lopinavir/ritonavir (iii) Chloroquine (iv) Ribavirin (v) IFN‐*α* (vi) Arbidol (vii) Intravenous immunoglobulin (viii) Traditional Chinese medicine	—	—	Length of stay	Length of stay
65	Yu et al., China	COVID‐19 patients benefit from early antiviral treatment: A comparative, retrospective study	27	(i) Arbidol (ii) Interferon (iii) Oseltamivir (iv) Ribavirin (v) Ganciclovir (vi) Antibiotic treatment (vii) Antifungal treatment (viii) Oxygen therapy (ix) Glucocorticoids (x) Immunotherapy	—	—	(i) Time from illness onset to be confirmed by SARS‐Cov‐2 RNA detection (ii) Time from illness onset to initiation of antiviral treatment (iii) Duration of total antiviral medication during the illness (iv) Time from illness onset to SARS‐CoV‐2 negative	(i) Time from illness onset to be confirmed by SARS‐Cov‐2 RNA detection (ii) Time from illness onset to SARS‐CoV‐2 negative
66	Llitjos et al., France	High incidence of venous thromboembolic events in anticoagulated severe COVID-19 patients	9	Prophylactic anticoagulation	Therapeutic anticoagulation 0.3–0.7 U/ml	—	(i) Acute respiratory distress syndrome (ii) Acute kidney injury (iii) Liver dysfunction (iv) Death	(i) Acute respiratory distress syndrome (ii) Death
67	Borie et al., France	Glucocorticoids with low-dose anti-IL1 anakinra rescue in severe non-ICU COVID-19 infection: A cohort study	42	(i) Corticosteroid ± anakinra (ii) Methylprednisolone 120 mg (daily dose) on three consecutive days (iii) Glucocorticoid	Thrombosis prophylaxis with LMWH. From February 15 to March 27, 2020	(i) Lopinavir-ritonavir (ii) Hydroxychloroquine (iii) Ivermectin, 12–15 mg (iv) Remdesivir (v) Thrombosis prophylaxis with LMWH (vi) Tocilizumab (vii) Corticosteroids, 120 mg (viii) Anakinra, 100 mg anakinra daily was added subcutaneously for ≤5days	(i) Death (ii) Invasive mechanical ventilation requirement within 15 days	(i) Death (ii) Invasive mechanical ventilation requirement within 15 days
68	Majmundar et al., the United States	Efficacy of corticosteroids in nonintensive care unit patients with COVID-19 pneumonia from the New York metropolitan region	57	(i) Corticosteroids (ii) Methylprednisolone (iii) Prednisone (iv) Dexamethasone	Noncorticosteroids	(i) Hydrocortisone (ii) Tocilizumab (iii) Enoxaparin therapeutic dose	(i) Intensive care unit (ICU) transfer (ii) ICU transfer (iii) Intubation (iv) Death (v) Discharge (vi) Length of stay	(i) Intensive care unit (ICU) transfer (ii) Intubation (iii) Death (iv) Length of stay
69	Martínez-Sanz et al., Spain	Effects of tocilizumab on mortality in hospitalized patients with COVID-19: A multicenter cohort study	24	Tocilizumab	Standard of care	(i) Corticosteroids (ii) Hydroxychloroquine (iii) Azithromycin (iv) Lopinavir/ritonavir	(i) Non-ICU length of stay (days) (ii) ICU admission (iii) ICU length of stay (days) (iv) Overall mortality (v) ICU mortality (vi) Non-ICU mortality (vii) ICU or mortality	(i) Non-ICU length of stay (days) (ii) ICU length of stay (days) (iii) Overall mortality (iv) ICU mortality (v) Non-ICU mortality (vi) ICU or mortality
70	Menzella et al., Italy	Efficacy of tocilizumab in patients with COVID-19 ARDS undergoing noninvasive ventilation	70	(i) Tocilizumab + standard therapy (ii) Hydroxychloroquine (iii) Antivirals (lopinavir/ritonavir or darunavir/cobicistat) (iv) Anticoagulants (full dosage) (v) Steroids (methylprednisolone 0.5–1 mg/kg/die)	(i) Standard therapy	(i) Hydroxychloroquine (ii) Antivirals (lopinavir/ritonavir or darunavir/cobicistat) (iii) Anticoagulants (full dosage) (iv) Steroids (methylprednisolone 0.5–1 mg/kg/die)	(i) Intubation/death (ii) Death	(i) Intubation/death (ii) Death
71	Mikulska et al, Italy	Tocilizumab and steroid treatment in patients with COVID-19 pneumonia	—	(i) Tocilizumab. 8 mg/kg (maximum 800 mg) (ii) Methylprednisolone. 1 mg/kg for 5 days intravenously, then 0.5 mg/kg for 5 days (iii)Standard of care	Standard of care	(i) Hydroxychloroquine. 400 mg bid (ii) Darunavir/ritonavir. 800/100 qd (iii) Low-molecular-weight heparin prophylaxis	(i) Time to failure, defined as intubation and mechanical ventilation or death (ii) Overall survival (iii) Time of hospitalization for the comparison between tocilizumab/methylprednisolone/SOC	(i) Time to failure, defined as intubation and mechanical ventilation or death (ii) Overall survival (iii) Time of hospitalization for the comparison between tocilizumab/methylprednisolone/SOC
72	Monreal et al., Spain	High versus standard doses of corticosteroids in severe COVID-19: A retrospective cohort study	61	High doses of corticosteroids: short-term pulse therapy of methylprednisolone-equivalent dosages from 250 to 1000 mg/day during one or more consecutive days.	Standard doses of corticosteroids. Methylprednisolone-equivalent dosages ranging from 0.5 to 1.5 mg/kg/day.	—	(i) Mortality (ii) A combined variable of need for mechanical or noninvasive MV and death. (iii) The development of severe ARDS, according to the Berlin definition	(i) Mortality (ii) A combined variable of need for mechanical or noninvasive MV and death
73	Pérez et al., Spain	Experience with tocilizumab in severe COVID-19 pneumonia after 80 days of follow-up: A retrospective cohort study	52	Tocilizumab: initial 600 mg, with a second or third dose (400 mg) in case of persistent or progressive disease	Not received tocilizumab	(i) Hydroxychloroquine (ii) Lopinavir/ritonavir (iii) Azithromycin.	(i) All-cause mortality (either in-hospital or after discharge) and associated factors. (ii) The impact of an early clinical response to tocilizumab in hospital and ICU stay. (iii) Evaluate safety of tocilizumab therapy.	(i) All-cause mortality (either in-hospital or after discharge) and associated factors. (ii) The impact of an early clinical response to tocilizumab in hospital and ICU stay. (iii) Evaluate safety of tocilizumab therapy.
74	Nadkarni et al., the United States	Anticoagulation, bleeding, mortality, and pathology in hospitalized patients with COVID-19	61	(i) Therapeutic anticoagulation (ii) Prophylactic anticoagulation	No anticoagulation	—	(i) Mortality (ii) Intubation and major bleeding	(i) Mortality (ii) Intubation and major bleeding
75	Nasir et al., Pakistan	Tocilizumab for COVID-19 acute respiratory distress syndrome: outcomes assessment using the WHO ordinal scale	121	Before tocilizumab	After tocilizumab	Concomitant steroids	(i) WHO ordinal scale (ii) Mortality (iii) Length of stay (iv) Development of nosocomial infection during hospitalization.	(i) WHO ordinal scale (ii) Mortality (iii) Length of stay
76	Omrani et al., Qatar	Convalescent plasma for the treatment of patients with severe coronavirus disease 2019: A preliminary report	62	Convalescent plasma	Standard of care	(i) Hydroxychloroquine (ii) Azithromycin (iii) lopinavir-ritonavir (iv) Tocilizumab (v) Methylprednisolone	(i) WHO ordinal scale (ii) Improvement in the respiratory status (iii) Discharged alive from ICU by study day 28 (iv) Viral clearance	(i) WHO ordinal scale (ii) Improvement in the respiratory status (iii) Discharged alive from ICU by study day 28 (iv)Viral clearance
77	Paccoud et al., France	Compassionate use of hydroxychloroquine in clinical practice for patients with mild to severe COVID-19 in a French university hospital	79	Hydroxychloroquine, 200 mg 3 times daily for 10 days	(i) Oxygen therapy to maintain an oxygen saturation >96% (ii) Intravenous or oral acetaminophen (iii) Antibiotics	—	(i) Death (ii) Admission to an ICU (iii) Time to death (iv) Time to hospital discharge for a return home or in an aftercare and rehabilitation (v) Adverse events recorded in patients receiving hydroxychloroquine treatment	(i) Death (ii) Admission to an ICU (iii) Adverse events recorded in the patients receiving hydroxychloroquine treatment
78	Price et al., the United States	Tocilizumab treatment for cytokine release syndrome in hospitalized patients with coronavirus disease 2019: survival and clinical outcomes	22	(i) Hydroxychloroquine (ii) Glucocorticoids (iii) Tocilizumab, 8 mg/kg intravenously, not to exceed 800mg;	—	—	(i) Survival (ii) 14 days survival (iii) Mechanical ventilation (iv) Days mechanically ventilated (v) Days of symptoms prior to hospitalization (vi) Days hospitalized (vii) Hospitalized at day 14	(i) Survival (ii) 14 days survival (iii) Mechanical ventilation (iv) Days mechanically ventilated (v) Days hospitalized (vi) Hospitalized at day 14
79	Rodríguez-Baño et al., Spain	Treatment with tocilizumab or corticosteroids for COVID-19 patients with hyperinflammatory state: A multicenter cohort study (SAM-COVID-19)	59	(i) Tocilizumab (ii) Corticosteroid pulse dose (iii) Corticosteroids intermediate-high dose (iv) Combination therapy	No treatment	-	(i) WHO ordinal scale (ii) Death or intubation	(i) WHO ordinal scale (ii) Death or intubation
80	Roomi et al., United States	Efficacy of hydroxychloroquine and tocilizumab in patients with COVID-19: single-center retrospective chart review	91	(i) Hydroxychloroquine (ii) Tocilizumab	(i) No hydroxychloroquine (ii) No tocilizumab	(i) Steroids (ii) Anticoagulation	(i) Invasive mechanical ventilation (ii) Mortality (iii) Discharge	(i) Invasive mechanical ventilation (ii) Mortality (iii) Dialysis
81	Antorán et al., Spain	Combination of tocilizumab and steroids to improve mortality in patients with severe COVID-19 infection: A Spanish, multicenter, cohort study	49	Tocilizumab	No tocilizumab	(i) Hydroxychloroquine (ii) Lopinavir/ritonavir (iii) Azithromycin (iv) Remdesivir (v) Interferon (vi) Steroids	Mortality	Mortality
82	Tortajada et al., Spain	Corticosteroids for COVID‐19 patients requiring oxygen support? Yes, but not for everyone: effect of corticosteroids on mortality and intensive care unit admission in patients with COVID‐19 according to patients' oxygen requirements	59	(i) Corticosteroids (ii) Methylprednisolone 250 mg iv once and 40 mg BIQ for 4 days (iii) Dexamethasone 20 mg iv QD for 5 days, followed by 10 mg QD for 5 more days	No corticosteroids	(i) Hydroxychloroquine (ii) Azithromycin (iii) Lopinavir/ ritonavir (iv) Tocilizumab (v) Interferon beta	(i) WHO ordinal scale. (ii) Admission to ICU or in‐hospital death (iii) Clinical improvement	(i) WHO ordinal scale. (ii) Admission to ICU or in‐hospital death (iii) Clinical improvement
83	Magagnoli et al., the United States	Outcomes of hydroxychloroquine usage in United States veterans hospitalized with COVID-19	21	(i) Hydroxychloroquine (ii) Hydroxychloroquine + azithromycin (iii) No hydroxychloroquine	—	—	(i) Mortality (ii) Use of mechanical ventilation	(i) Mortality (ii) Use of mechanical ventilation
84	Joyner et al., United States	Early safety indicators of COVID-19 convalescent plasma in 5000 patients	39	Convalescent plasma	—	—	The safety of transfusion of COVID-19 convalescent plasma assessed as the incidence and relatedness of severe adverse events including death.	The safety of transfusion of COVID-19 convalescent plasma assessed as the incidence and relatedness of severe adverse events including death.
85	Rajter et al., South Florida, the United States	Use of ivermectin is associated with lower mortality in hospitalized patients with coronavirus disease 2019: The ivermectin in COVID-19 study	58	Ivermectin 200 *μ*g/kg	No ivermectin	(i) Corticosteroid (ii) Hydroxychloroquine (iii) Azithromycin	(i) Mortality (ii) Successful extubation (iii) Length of hospital stay	(i) Mortality (ii) Successful extubation (iii) Length of hospital stay.
86	Hanif et al., the United States	Thrombotic complications and anticoagulation in COVID-19 pneumonia: A New York City hospital experience	31	(i) Therapeutic anticoagulation prior to admission (ii) Therapeutic anticoagulation during the admission (iii) Prophylactic anticoagulation only during the hospital stay (iv) No anticoagulation	—	—	(i) Mortality (ii) Length of stay (iii) Intubation (iv) Successful extubation	(i) Mortality (ii) Length of stay (iii) Intubation (iv) Successful extubation
87	Duan et al., China	Effectiveness of convalescent plasma therapy in severe COVID-19 patients	30	Convalescent plasma: one dose of 200 ml of inactivated CP with neutralization activity of >1:640 was transfused into the patients within 4 h following the WHO blood transfusion protocol.	—	(i) Antiviral therapy (ii) Other supportive care (iii) Antibiotic treatment (iv) Antifungal treatment (v) Glucocorticoid (vi) Oxygen support at the appropriate situation	(i) The safety of convalescent plasma transfusion. (ii) The improvement of clinical symptoms and laboratory and radiological parameters within 3 days after (iii) Convalescent plasma transfusion.	(i) The safety of convalescent plasma transfusion. (ii) The improvement of clinical symptoms and laboratory and radiological parameters within 3 days after (iii) Convalescent plasma transfusion.

**Table 3:** Description of study characteristics: case-control studies.

S. no.	Author, country	Title	Study duration in days	Intervention group	Control group	Concomitant intervention	Outcome measures	Outcome measures applicable to this review
1	Klopfenstein et al., France	Impact of tocilizumab on mortality and/or invasive mechanical ventilation requirement in a cohort of 206 COVID-19 patients	72	Tocilizumab 8 mg/kg per dose, 1 or 2 doses	(i) Standard treatment (ii) Hydroxychloroquine (iii) Lopinavir-ritonavir therapy (iv) Antibiotics (v) Corticosteroids	(i) Hydroxychloroquine (ii) Lopinavir-ritonavir therapy (iii) Antibiotics (iv) Corticosteroids	(i) Mortality (ii) Intensive mechanical ventilation (iii) Duration to hospitalization	(i) Mortality (ii) Intensive mechanical ventilation (iii) Duration to hospitalization
2	Klopfenstein et al., France	Tocilizumab therapy reduced intensive care unit admissions and/or mortality in COVID-19 patients	24	Tocilizumab 8 mg/kg per dose, 1 or 2 doses	(i) Standard treatment (ii) Hydroxychloroquine (iii) Lopinavir-ritonavir therapy (iv) Antibiotics (v) Corticosteroids	(i) Hydroxychloroquine (ii) Lopinavir-ritonavir therapy (iii) Antibiotics (iv) Corticosteroids	(i) Death (ii) ICU admission (iii) Invasive mechanical ventilation (iv) Duration of hospitalization	(i) Death (ii) ICU admission (iii) Invasive mechanical ventilation (iv) Duration of hospitalization
3	Sean et al., the United States	Convalescent plasma treatment of severe COVID-19: a propensity score-matched control study	16	Convalescent plasma therapy	—	(i) Azithromycin (ii) Hydroxychloroquine (iii) Broad-spectrum antibiotics (iv) Therapeutic dose anticoagulation (v) Corticosteroids (vi) Remdesivir (vii) Mesenchymal stem cells and interleukin (IL)-1 and IL-6 inhibitors	(i) Survival (ii) Oxygen requirement	(i) Survival (ii) Oxygen requirement
4	Abolghasemi et al., Iran	Clinical efficacy of convalescent plasma for treatment of COVID-19 infections: results of a multicenter clinical study	61	Convalescent plasma 500 cc (one unit)	No convalescent plasma	(i) Lopinavir/ritonavir (ii) Hydroxychloroquine	(i) Mortality (ii) Intubation (iii) Length of stay (iv) Improvements in clinical symptoms (v) Adverse events from treatment	(i) Mortality (ii) Intubation (iii) Length of stay (iv) Improvements in clinical symptoms (iv) Adverse events from treatment
5	Rossotti et al., Italy	Safety and efficacy of anti-IL-6 receptor tocilizumab use in severe and critical patients affected by coronavirus disease 2019: A comparative analysis	—	Tocilizumab	(i) Hydroxychloroquine plus lopinavir/ritonavir (ii) Remdesivir	—	(i) Survival (ii) Length of stay	(i) Survival (ii) Length of stay
6	Matthieu et al., France	Clinical efficacy of hydroxychloroquine in patients with COVID-19 pneumonia who require oxygen: observational comparative study using routine care data	43	Hydroxychloroquine	No hydroxychloroquine	(i) Azithromycin (ii) Amoxicillin (iii) Tocilizumab (iv) Lopinavir-ritonavir (v) Remdesivir	(i) Survival (ii) Weaning from oxygen	(i) Survival (ii) Weaning from oxygen
7	Perrone et al., Italy	Tocilizumab for patients with COVID-19 pneumonia: The single-arm TOCIVID-19 prospective trial	34	Tocilizumab 8 mg/kg up to a maximum of 800 mg per dose	—	(i) Antiretroviral (ii) Hydroxy chloroquine (iii) Antibiotics (iv) Steroids (v) Low-molecular-weight heparin	Lethality rate	Lethality rate
8	G. Rojas-Marte et al., the United States	Outcomes in patients with severe COVID-19 disease treated with tocilizumab: a case-controlled study	49	Tocilizumab	(i) Hydroxychloroquine (ii) Azithromycin (iii) Corticosteroids (iv) Anticoagulation (v) Remdesivir (vi) Antibiotics (vii) Vasopressors	(i) Hydroxychloroquine (ii) Azithromycin (iii) Corticosteroids (iv) Anticoagulation (v) Remdesivir (vi) Antibiotics (vii) Vasopressors	(i) Overall mortality rate (ii) Mortality in nonintubated patients only (iii) Mortality in intubated patients (iv) Length of stay	(i) Overall mortality rate. (ii) Mortality in nonintubated patients only (iii) Mortality in intubated patients (iv) Length of stay
9	Scarsi et al., Italy	Association between treatment with colchicine and improved survival in a single-center cohort of adult hospitalized patients with COVID-19 pneumonia and acute respiratory distress syndrome	32	(i) Colchicine 1 mg/day (ii) Standard of care (hydroxychloroquine, lopinavir/ritonavir, and intravenous dexamethasone)	Standard of care (hydroxychloroquine, lopinavir/ritonavir, and intravenous dexamethasone)	—	Survival rate	Survival rate
10	Keller et al., The Bronx	Effect of systemic glucocorticoids on mortality or mechanical ventilation in patients with COVID-19	34	Early glucocorticoid first 48 hours	No glucocorticoid	—	(i) In-hospital mortality (ii) In-hospital mechanical ventilation. (iii) Mortality in mechanical ventilation	(i) In-hospital mortality (ii) In-hospital mechanical ventilation. (iii) Mortality in mechanical ventilation
11	Yu et al., China	Lopinavir/ritonavir is associated with pneumonia resolution in COVID-19 patients with influenza coinfection: A retrospective matched-pair cohort study	30	Lopinavir/ritonavir treatment	No lopinavir/ritonavir treatment	(i) Glucocorticoid treatment (ii) Ribavirin treatment (iii) Lopinavir/ritonavir treatment (iv) Oseltamivir (v) Arbidol	(i) Dead or deteriorated (ii) Cured	(i) Dead or deteriorated (i) Cured
12	Qu et al., not mentioned	Comparative effectiveness of lopinavir/ritonavir-based regimens in COVID-19	—	(i) Lopinavir/ritonavir (LPV/r) alone (ii) Lopinavir/ritonavir (LPV/r) + Novaferon (iii) Lopinavir/ritonavir (LPV/r) + interferon (iv) Lopinavir/ritonavir (LPV/r) + interferon + Novaferon (v) Lopinavir/ritonavir (LPV/r) + interferon + Arbidol (LPV/r: PO 500 mg (400 mg lopinavir + 100 mg ritonavir) BID; Novaferon: aerosol 20 microgram BID; Arbidol: PO 0.2 g TID; interferon: aerosol 500 × 104 IU·BID)	—	—	(i) Time of negative nucleic acid conversion. (ii) Length of hospitalization (iii) The rate of adverse reaction (iv) Transferring to ICU and clinical mechanical therapy	(i) Time of negative nucleic acid conversion. (ii) Length of hospitalization. (iii) The rate of adverse reaction (iii) Transferring to ICU and clinical mechanical therapy

**Table 4:** Description of study characteristics: interventional studies.

S. no.	Trial registration number	Author, country	Title	Study duration in days	Study arm	Intervention group	Control group	Concomitant drugs	Outcome measures	Outcome measures applicable to this review
1	NCT04353336	Abd-Elsalam et al., Egypt	Hydroxychloroquine in the treatment of COVID-19: A multicenter randomized controlled study	122	2	Hydroxychloroquine	(i) Paracetamol (ii) Oxygen (iii) Fluids (iv) Empiric antibiotic (cephalosporins) (v) Oseltamivir (vi) Invasive mechanical ventilation with hydrocortisone	—	(i) Death (ii) Duration of hospital stay	(i) Death (ii) Duration of hospital stay
2	Trial registration not specified.	Antinori et al., Italy	Compassionate remdesivir treatment of severe COVID-19 pneumonia in intensive care unit (ICU) and non-ICU patients: clinical outcome and differences in posttreatment hospitalization status	27	1	Remdesivir (ICU and ward setting)	None	—	(i) WHO ordinal scale (ii) Hospitalization status (iii) Adverse events	(i) WHO ordinal scale (ii) Hospitalization status (iii) Adverse events
3	NCT04323527	Borba et al., Brazil	Effect of high vs. low doses of chloroquine diphosphate as adjunctive therapy for patients hospitalized with severe acute respiratory syndrome coronavirus 2 (SARS-CoV-2) infection	—	2	High-dose chloroquine (600 mg CQ; 4 × 150 mg tablets twice daily for 10 days; total dose 12 g)	Low-dose chloroquine (450 mg CQ twice daily on the first day and 450 mg once daily for 4 days)	(i) Intravenous ceftriaxone (1 g twice daily for 7 days) (ii) Azithromycin (500 mg once daily for 5 days) (iii) Oseltamivir (75 mg twice daily for 5 days)	(i) Safety (ii) Lethality (iii) Clinical status (iv) Laboratory examinations (v) Electrocardiogram results	(i) Safety (ii) Lethality (iii) Clinical status
4	ChiCTR2000029308	Cao et al., China	A trial of lopinavir-ritonavir in adults hospitalized with severe COVID-19	17	2	Lopinavir-ritonavir (400 mg and 100 mg twice daily)	(i) Supplemental oxygen (ii) Noninvasive ventilation (iii) Invasive ventilation (iv) Antibiotic agents (v) Vasopressor support (vi) Renal replacement therapy (vii) Extracorporeal membrane oxygenation (ECMO)	—	(i) WHO ordinal scale (ii) Time to clinical improvement (iii) Day 28 mortality (iv) ICU length of stay	(i) WHO ordinal scale (ii) Time to clinical improvement (iii) Day 28 mortality (iv) ICU length of stay
5	IRCT20200501047259N1	Gharebaghi et al., Iran	The use of intravenous immunoglobulin gamma for the treatment of severe coronavirus disease 2019: A randomized placebo-controlled double-blind clinical trial	—	2	Intravenous immunoglobulin (IVIG). Four vials of 5 g IVIG daily	Placebo and standard of care	—	Mortality	Mortality
6	NCT04383535	Simonovich et al., Italy	A randomized trial of convalescent plasma in COVID-19 severe pneumonia	—	2	Convalescent plasma	Placebo and standard of care	(i) Antiviral agents (ii) Glucocorticoids	(i) WHO ordinal scale (ii) Clinical status at 30 days (iii) 30 days mortality	(i) WHO ordinal scale (ii) Clinical status at 30 days (iii) 30 days mortality
7	NCT04375098	Balcells et al., Chile	Early anti-SARS-CoV-2 convalescent Plasma in patients admitted for COVID-19: A randomized phase II clinical trial	70	2	Early plasma, 400 ml of ABO compatible convalescent plasma	Deferred plasma, 400 ml plasma	(i) Steroids (ii) Tocilizumab (iii) Hydroxychloroquine (iv) Lopinavir/ritonavir (v) Thromboprophylaxis (vi) Anticoagulation	(i) Mechanical ventilation (ii) Hospitalization >14 days (iii) Death (iv) Oxygen requirement	(i) Mechanical ventilation (ii) Hospitalization >14 days (iii) Death (iv) Oxygen requirement
8	IRCT20150303021315N17	Malekzadeh et al., Iran	Subcutaneous tocilizumab in adults with severe and critical COVID-19: A prospective open-label uncontrolled multicenter trial	100	1	Tocilizumab at a dose of 324 mg	(i) Antiviral agents (ii) Hydroxychloroquine (iii) Interferon beta-1a (iv) Antibiotic agents	—	(i) Hospital Stay (ii) Death (iii) Oxygen requirement (iv) Adverse events	(i) Hospital stay (ii) Death (iii) Oxygen requirement (iv) Adverse events
9	NCT04346355	Salvarani et al., Italy	Effect of tocilizumab vs. standard care on clinical worsening in patients hospitalized with COVID-19 pneumonia: A randomized clinical trial	73	2	Tocilizumab at a dose of 8 mg/kg up to a maximum of 800 mg	(i) Tocilizumab IV + steroids (ii) Steroids (iii) Canakinumab	(i) Hydroxychloroquine (ii) Heparin (iii) LMWH (iv) Antiretroviral (v) azithromycin	(i) Clinical worsening (ii) At 14 days: admissions to ICU (iii) At 14 days: deaths (iv) At 14 days: discharges (v) At 30 days: admissions to ICU (vi) At 30 days: deaths (vii) At 30 days: discharges	(i) Clinical worsening (ii) At 14 days: admissions to ICU (iii) At 14 days: deaths (iv) At 14 days: discharges (v) At 30 days: Admissions to ICU (vi) At 30 days: deaths (vii) At 30 days: discharges
10	NCT04356937	Stone et al., the United States	Efficacy of tocilizumab in patients hospitalized with COVID-19	57	2	Tocilizumab, 8 mg per kilogram of bodyweight administered intravenously, not to exceed 800 mg	Placebo and standard of care	(i) Remdesivir (ii) Dexamethasone (iii) Hydroxychloroquine (iv) Glucocorticoids	(i) Death (ii) Intubation (iii) Oxygen requirement	(i) Death (ii) Intubation (iii) Oxygen requirement
11	NCT04257656	Wang et al., China	Remdesivir in adults with severe COVID-19: a randomized, double-blind, placebo-controlled, multicenter trial	36	2	Remdesivir, 200 mg on day 1 followed by 100 mg on days 2–10 in single daily infusions	Placebo and standard of care	(i) Lopinavir-ritonavir (ii) Interferons (iii) Corticosteroids	(i) Time to clinical improvement within 28 days (ii) Mortality at day 28 (iii) Safety outcomes included treatment-emergent adverse events, serious adverse events, and premature discontinuation of study drugs.	(i) Time to clinical improvement within 28 days (ii) Mortality at day 28 (iii) Safety outcomes included treatment-emergent adverse events, serious adverse events, and premature discontinuation of study drugs.
12	NCT04405843	Medina et al., Colombia	Effect of ivermectin on time to resolution of symptoms among adults with mild COVID-19: A randomized clinical Trial	17	2	Ivermectin and standard of care: received 300 *μ*g/kg	Placebo and standard of care	(i) NSAIDS (ii) Macrolides (iii) Antipyretics (iv) Antibiotics (v) Glucocorticoids (vi) Immunomodulating (vii) Anticoagulants	(i) Deaths (ii) Clinical deterioration (iii) Adverse events	(i) WHO ordinal scale (ii) Deaths (iii) Clinical deterioration (iv) Adverse events
13	NCT04276688	Hung et al., Hong Kong	Triple combination of interferon beta-1b, lopinavir-ritonavir, and ribavirin in the treatment of patients admitted to hospital with COVID-19: An open-label, randomized, phase 2 trial	40	2 arms	(i) Interferon beta-1b (ii) Lopinavir-ritonavir (iii) Ribavirin	Lopinavir-ritonavir	—	(i) Mortality (ii) Length of hospital stay (iii) Negative RT-PCR result	(i) Mortality (ii) Length of hospital stay (iii) Negative RT-PCR result
14	ChiCTR2000029387	Huang et al., China	No statistically apparent difference in antiviral effectiveness observed among ribavirin plus interferon-alpha, lopinavir/ritonavir plus interferon-alpha, and ribavirin plus lopinavir/ritonavir plus interferon-alpha in patients with mild-to-moderate coronavirus disease 2019: results of a randomized, open-labelled prospective study	28	3	(i) Ribavirin (ii) Lopinavir/ritonavir (iii) Interferon-alpha	—	—	(i) Death (ii) Length of hospitalization (iii) Negative SARS-CoV-2 results (iv) Adverse events	(i) Death (ii) Length of hospitalization (iii) Negative SARS-CoV-2 results (iv) adverse events
15	NCT04292899	Olender et al., multicenter	Remdesivir for severe coronavirus disease 2019 (COVID-19) versus a cohort receiving standard of care	33	2	Remdesivir	No remdesivir	(i) Azithromycin (ii) Hydroxychloroquine (iii) HIV protease inhibitor (iv) Biologics (v) Ribavirin	(i) WHO ordinal score (ii) Recovery on day 14 (iii) Death at day 14 (iv) Clinical improvement on day 14	(i) WHO ordinal score (ii) Recovery on day 14 (iii) Death at day 14 (iv) Clinical improvement on day 14
16	ChiCTR2000029757	Li et al., China	Effect of convalescent plasma therapy on time to clinical improvement in patients with severe and life-threatening COVID-19: A randomized clinical trial	48	2	Convalescent plasma	Standard of care	(i) Antiviral (ii) Antibacterial (iii) Steroids (iv) Human immunoglobulin	(i) WHO ordinal scale (ii) Time to clinical improvement (iii) Discharge rate at 28 days (iv) Mortality at 28 days (v) Negative PCR	(i) WHO ordinal scale (ii) Time to clinical improvement (iii) Discharge rate at 28 days (iv) Mortality at 28 days (v) Negative PCR
17	NCT 04321421	Perotti et al., Italy	Mortality reduction in 46 severe COVID-19 patients treated with hyperimmune plasma: A proof-of-concept single arm multicenter interventional trial	32	1	Plasma infusion	None	(i) Antiviral (ii) Antibiotics (iii) Hydroxychloroquine (iv) Anticoagulant	(i) Mortality (ii) Changes in PaO_2_/FiO_2_, LDH	(i) Mortality (ii) Oxygen requirement
18	NCT04292730	Spinner et al., multicenter	Effect of remdesivir vs standard care on clinical status at 11 days in patients with moderate COVID-19: A randomized clinical trial	35	3	10 days remdesivir	(i) 5 days remdesivir (ii) Standard of care	(i) Steroids (ii) Hydroxychloroquine/chloroquine (iii) Lopinavir-ritonavir (iv) Tocilizumab (v) Azithromycin	(i) Death (ii) Discharge (iii) Adverse events	(i) Death (ii) Discharge (iii) Adverse events
19	CTRI/2020/04/024775	Agarwal et al., India	Convalescent plasma in the management of moderate COVID-19 in adults in India: open-label phase II multicenter randomized controlled trial (PLACID trial)	84	2	Convalescent plasma (two doses of 200 ml) + best standard of care.	Standard of care	(i) Hydroxychloroquine (ii) Remdesivir (iii) Lopinavir (iv) Ritonavir (v) Oseltamivir (vi) Antibiotics (vii) Steroids (viii) Tocilizumab	(i) Mortality (ii) Clinical improvement on the World Health Organization ordinal scale	(i) Mortality (ii) Clinical improvement on the World Health Organization ordinal scale

**Table 5:** Description of study characteristics: quasiexperimental studies.

S. no.	Trial registration number	Author, country	Title	Study duration in days	Study arm	Intervention group	Control group	Concomitant interventions	Outcome measures	Outcome measures applicable to this review
1	NCT04374071	Fadel et al., United States	Early short-course corticosteroids in hospitalized	8	2	Corticosteroid methylprednisolone 0.5–1 mg/kg/day divided in 2 intravenous doses for 3 days	(i) Standard care (ii) Lopinavir-ritonavir (iii) Ribavirin (iv) Hydroxychloroquine (v) Steroid	(i) Lopinavir-ribavirin (ii) Hydroxychloroquine (iii) Tocilizumab (iv) Methylprednisolone (v) Oral prednisone	(i) Death (ii) Respiratory failure requiring mechanical ventilation. (iii) Overall mechanical ventilation (iv) Length of stay	(i) Death (ii) Respiratory failure requiring mechanical ventilation. (iii) Overall mechanical ventilation (iv) Length of stay
2	NCT04374071	Fatima et al., Pakistan	Comparison of efficacy of dexamethasone and methylprednisolone in moderate to severe COVID-19 disease.	30	2	Intravenous methylprednisolone 1 mg/kg/day in 2 divided	Intravenous dexamethasone 8 mg/day given for 5 days	(i) Plasma therapy (ii) Antibiotics (iii) Tocilizumab	(i) Mortality (ii) ICU transfer (iii) Ventilator needed	(i) Mortality (ii) ICU transfer (iii) Ventilator needed
3	NCT4357106	Olivares-Gazca et al., Mexico	Infusion of convalescent plasma is associated with clinical improvement in critically ill patients with COVID-19: A pilot study	22	1	Convalescent plasma	—	—	Mortality	Mortality
4	2020-000890-25	Philippe Gautret et al., France	Hydroxychloroquine and azithromycin as a treatment of COVID-19: results of an open-label nonrandomized clinical trial	—	2	Hydroxychloroquine 200 mg, 3 times per day for 10 days	No hydroxychloroquine	(i) Azithromycin 500 mg on day 1 and 250 mg per day for the next four days (hydroxychloroquine-treated patients) (ii) Combination of hydroxychloroquine and azithromycin	(i) Virological clearance at day 6 (ii) Virological clearance over the time (iii) Occurrence of side effects	(i) Virological clearance at day 6 (ii) Virological clearance over the time (iii) Occurrence of side effects

**Table 6:** Comparison of recommendations.

S. no.	Intervention	Recommendations		
	Drugs	National guidelines	Systematic review	WHO
1	Corticosteroids	(i) To use in severe or critical patients (ii) Not to use in nonsevere or asymptomatic	(i) For the use of corticosteroids in severe and critical patients, hospitalized COVID-19 patients. Strong recommendation, moderate-quality evidence (ii) Against the use of corticosteroids in nonsevere patients, hospitalized COVID-19 patients. Weak recommendation, moderate-quality evidence	Recommended the use of systematic corticosteroid rather than no corticosteroids in severe and critical COVID-19 patients.
2	Tocilizumab	(i) To use in patients who have worsened despite the initial 24–48 hours of steroids (ii) To not use in patients who have not received a trial of steroids or with elevated markers only	For the use of tocilizumab in hospitalized COVID-19 patients. Weak recommendation, moderate-quality evidence	Recommended the use of tocilizumab in patients with severe or critical COVID-19 infection.
3	Ivermectin therapy	This is not recommended in the national guidelines	Against the use of ivermectin therapy in the use of COVID-19 hospitalized patients. Weak recommendation, low-quality evidence	Recommended against the use of ivermectin in patients with COVID-19
4	Hydroxychloroquine/chloroquine	There is no role for prophylactic chloroquine and hydroxychloroquine to prevent COVID-19 infection after exposure	Against the use of hydroxychloroquine alone or in combination with other antibiotics in hospitalized COVID-19 patients. Weak recommendation, moderate-quality evidence	Recommended against the use of hydroxychloroquine or chloroquine for treatment of COVID-19
5	Antibiotics	(i) To use in proven or strong suspicion of secondary infection (ii) To not use for “prevention” of secondary infections or in patients with no clear evidence of bacterial infection	No evidence available	No evidence available
6	Anticoagulation therapy	Prophylactic anticoagulation (i) To use in all hospitalized patients (ii) To not use in nonsevere or asymptomatic patients Therapeutic anticoagulation (i) To use in proven or high suspicion of VTE (ii) To not use in patients with isolated elevated D-dimers or no evidence of VTE	(i) For the use of anticoagulant therapeutic doses. Therapeutic: strong recommendation, moderate-quality evidence (ii) For the use of prophylactic dose anticoagulants to treat COVID-19 hospitalized patients. Prophylactic: strong recommendation, moderate-quality evidence	No evidence available
7	Remdesivir	(i) To use in severe patients with less than 10 days of symptoms (ii) To not use in nonsevere, asymptomatic, or critical patients or in whom symptoms are longer than 10 days	For the use of remdesivir in hospitalized COVID-19 patients. Recommendation, high-quality evidence	Conditional recommendation against administering remdesivir in addition to usual care.
8	Lopinavir/ritonavir	(i) To use in severe patients with less than 10 days of symptoms (ii) To not use in nonsevere, asymptomatic, or critical patients or in whom symptoms are longer than 10 days	Against the use of ritonavir/lopinavir in hospitalized COVID-19 patients. No recommendation, moderate-quality evidence	Recommended against administering lopinavir/ritonavir for treatment of COVID-19.
9	Convalescent plasma	No evidence available	Against the use of convalescent plasma in the management of hospitalized COVID-19 patients. Weak recommendation, moderate-quality evidence	No evidence available
10	Famotidine	Not recommended in the national guidelines	For the use of famotidine in hospitalized COVID-19 patients. Weak recommendation, low-quality evidence	No evidence available
11	Immunoglobulin therapy	No evidence available	For the use of immunoglobulin therapy in hospitalized COVID-19 patients. Weak recommendation, moderate-quality evidence	No evidence available
12	Colchicine	No evidence available	Against the use of colchicine in hospitalized COVID-19 patients. No recommendation, low-quality evidence	No evidence available

## Data Availability

The data used to support the findings of this study are available from the corresponding author upon request.

## Supplementary Materials

Supplementary Materials Supplementary Figure 1. PRISMA flow diagram reporting various studies assessed for further evaluation and included in the review. Supplementary Figure 2. Risk of bias graph for quasiexperimental studies. Supplementary Figure 3. Risk of bias summary for quasiexperimental studies. Supplementary Figure 4. Risk of bias graph for randomized control trials. Supplementary Figure 5. Risk of bias summary for randomized control trials. Supplementary Figure 6. Risk of bias graph for case-control studies. Supplementary Figure 7. Risk of bias summary for case-control studies. Supplementary Figure 8. Risk of bias graph for observational cohort and cross-sectional Studies. Supplementary Figure 9. Risk of bias summary for observational cohort and cross-sectional studies.
