# Supplementary material for: Development of Evidence-Based COVID-19 Management Guidelines for Local Context: The Methodological Challenges
Source: Glob Health Epidemiol Genom. 2022 Apr 20;2022:4240378. doi: 10.1155/2022/4240378 (PMC9020141; doi:10.1155/2022/4240378)
Supplement: Supplementary Materials — Supplementary Figure 1. PRISMA flow diagram reporting various studies assessed for further evaluation and included in the review. Supplementary Figure 2. Risk of bias graph for quasiexperimental studies. Supplementary Figure 3. Risk of bias summary for quasiexperimental studies. Supplementary Figure 4. Risk of bias graph for randomized control trials. Supplementary Figure 5. Risk of bias summary for randomized control trials. Supplementary Figure 6. Risk of bias graph for case-control studies. Supplementary Figure 7. Risk of bias summary for case-control studies. Supplementary Figure 8. Risk of bias graph for observational cohort and cross-sectional Studies. Supplementary Figure 9. Risk of bias summary for observational cohort and cross-sectional studies. [file 4240378.f1.docx]

Records identified through database searching. (CINAHL= 4525
WHO= 8968
PubMed=5175
Clinical Trial=5016
Google Scholar= 936
Total= 24620)

Duplicates removed (n=1333)

**Identification**

Records screened (n=23287)

Records excluded titles and abstracts.
(n=22485)

**Screening**

Full text articles excluded (n=680):

- Focus on chemical composition of drugs=11
- Laboratory findings=12
- No treatment specified = 6
- Studies not included hospitalized adult COVID-19 patients =151
- Study Protocol = 44
- *Other than RCTs and Observational studies= 98
- Grey Literature = 95
- **Others = 263

Full-text articles assessed for eligibility (n=802)

**Eligibility**

Studies included for Extraction.
Total: 122
(87 cohort/cross-sectional studies, 19 interventional studies/randomized controlled trials, 12 case-control studies, and 4 quasi-experimental studies)

**Included**

**(Supplementary Figure. 1): PRISMA flow diagram reported various studies assessed for further evaluation and including in the review**
